# IL-6 Does Not Influence the Expression of *SLC41A1* and Other Mg-Homeostatic Factors

**DOI:** 10.3390/ijms252413274

**Published:** 2024-12-11

**Authors:** Maria Brodnanova, Michal Cibulka, Marian Grendar, Eduard Gondas, Martin Kolisek

**Affiliations:** 1 Biomedical Centre Martin, Jessenius Faculty of Medicine in Martin, Comenius University Bratislava, 03601 Martin, Slovakia; maria.brodnanova@uniba.sk (M.B.); michal.cibulka@uniba.sk (M.C.); marian.grendar@uniba.sk (M.G.); 2 Department of Pharmacology, Jessenius Faculty of Medicine in Martin, Comenius University Bratislava, 03601 Martin, Slovakia; eduard.gondas@uniba.sk

**Keywords:** magnesium, inflammation, interleukin-6, STAT3, *SLC41A1*

## Abstract

Together with chronic inflammation, disturbed magnesium homeostasis is a factor accompanying chronic disease which thus contributes to a reduced quality of human life. In this study, our objective was to examine the possible IL-6-mediated chronic inflammation-dependent regulation of nine magnesiotropic genes encoding for constituents of magnesium homeostasis of the cell. We used three cell lines (HepG2, U-266, and PANC-1), all characterized by high expression of the *IL6R* gene and the presence of a membrane form of IL-6R capable of responding to human IL-6. Despite the confirmed activation of the IL-6R/JAK/STAT3 pathway after hIL-6 treatment, we observed no biologically relevant changes in the transcription intensity of the studied magnesiotropic genes. This, however, does not exclude the possibility that IL-6 can affect magnesium homeostasis at levels other than through modified transcription.

## 1. Introduction

Disturbed magnesium (Mg) homeostasis in cells and at the level of the entire body is recognized as an important factor contributing to the onset of various diseases, many of which are accompanied by chronic inflammation. Systemic hypomagnesemia and cellular Mg insufficiency is correlated with pathological conditions such as diabetes mellitus type 2 (DMT2), hypertension, or obesity [1,2].

Approximately 50% of Americans obtain insufficient Mg in their regular diet, despite a wealth of research supporting the mineral’s importance for human health [3,4]. A serum [Mg] of less than 0.85 mmol/L, which is regarded to reflect a systemic subclinical Mg shortage [5], may cause or exacerbate chronic inflammation when combined with other risk factors [1,2]. A diet low in Mg and hypomagnesemia are inversely correlated with elevated serum levels of C-reactive protein (CRP), which serves as an early marker of the inflammatory process in the body [6]. CRP is a routinely used marker of the acute phase of the inflammatory process, for example in infectious diseases, but its elevated concentration in the circulation is also associated with chronic diseases such as DMT2, cardiovascular disease, or rheumatoid arthritis [7,8]. On the other hand, Mg supplementation leads to the suppression of markers correlated with the inflammatory process [9,10,11,12,13].

CRP is secreted into the bloodstream by the liver, where it is synthesized by hepatocytes under the control of interleukin-6 (IL-6) [14]. IL-6 is a cytokine whose concentration in the circulation is also significantly elevated during inflammation [15,16]. However, IL-6 is considered to possess both pro-inflammatory and anti-inflammatory effects. Its anti-inflammatory activity is associated with the classic signaling pathway, which requires the membrane form of both parts of the IL-6 receptor (IL-6R) subunits, namely IL-6Rα (encoded by *IL6R*) and 130 kDa glycoprotein (gp130) (encoded by *IL6ST*) [17]. On the other hand, IL-6 exhibits pro-inflammatory properties in the presence of a soluble form of the α-subunit, specifically sIL-6R, which in complex with IL-6 can bind directly with gp130 in the absence of the IL-6Rα membrane form. This mechanism of activation is known as the trans-signaling pathway [17].

The binding of IL-6 to the membrane (IL-6R) or soluble (sIL-6R) α-subunit triggers the homodimerization of gp130 molecules. This event leads to the activation of Janus kinase (JAK), which subsequently phosphorylates the signal transducer and activator of transcription 3 (STAT3) at position Y^705^ [18]. After STAT3 phosphorylation and dimerization, the STAT3 homodimer enters the nucleus, where it acts as a transcription factor [18].

The activated gp130 receptor also initiates the MAPK and PI3K pathways [19]. In addition to the phosphorylation of Y^705^, the S^727^ site undergoes phosphorylation by PKC kinase activity (upon IL-6 stimulation [20]), JNK1 [21], or ERK [22]. This serine residue phosphorylation might be required for the full transcription activity of STAT3 [23] and is subsequently associated with the potentiation of STAT3 inactivation [24,25]. Phosphorylation at S^727^ stabilizes STAT3′s interaction with the transcriptional coactivator CBP/p300 [26], enabling further modifications. Namely its acetylation at the K^685^ site by CBP/p300 histone acetyltransferase activity triggered by the PI3K/Akt pathway. K^685^ acetylation further increases STAT3 stability [27].

**Figure 1:**
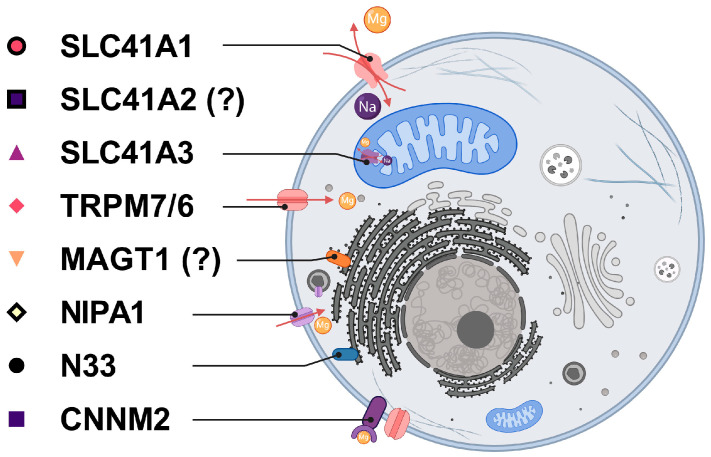
Current understanding of the cellular localization of Mg^2+^-transport proteins and other Mg-homeostatic factors in the eukaryotic cell. Na^+^/Mg^2+^ exchanger SLC41A1 facilitates the export of Mg^2+^ from the cell in exchange for Na^+^ [32,33]. SLC41A3 mediates this exchange on the inner mitochondrial membrane [35]. TRPM6/7 are channels that facilitate the entry of multiple divalent cations through the plasma membrane of the cell [28,30,36]. MAGT1 and N33 are part of the N-glycosylation machinery of the cell and likely contribute to Mg homeostasis indirectly [37,38]. NIPA1 facilitates the transport of Mg^2+^ across the plasma membrane [39]. CNNM2 regulates Mg-homeostasis more through the regulation of other Mg-homeostatic factors rather than directly facilitating its transport [40]. The function and precise cellular localization of SLC41A2 have not yet been fully elucidated, despite its high homology to SLC41A1 and SLC41A3 [41].

The pool of intracellular free ionized Mg (Mg^2+^) is regulated by the entry of Mg^2+^, its extrusion, and/or its mobilization of intracellular Mg^2+^ stores (Figure 1). The main pathway of cellular Mg^2+^ uptake is through a ubiquitously expressed divalent cation chanzyme, namely transient receptor potential melastatin type 7 (TRPM7) [28]. The TRPM7 homologue, TRPM6, is relatively well studied and is similar to TRPM7 in that it also possesses both the ion channel and protein kinase activities [29]. However, because TRPM6 plays a crucial function in controlling the body’s Mg homeostasis by taking part in the absorption of Mg^2+^ in the intestine and its resorption in the kidney, it is mostly expressed in the epithelial cells of the colon and kidney [30,31]. The regulated extrusion of Mg^2+^ occurs primarily through the plasma membrane Na^+^/Mg^2+^ exchanger solute carrier family 41 member 1 (SLC41A1) [32,33], which is encoded by the gene located at the *PARK16* locus, a locus associated with susceptibility to neurodegenerative Parkinson’s disease [34]. Its homologue SLC41A3 is a mitochondrial Na^+^-dependent Mg^2+^ efflux system [35].

Despite several studies [9,12,42] having established an association between Mg status and *IL6* expression, the regulation of Mg homeostasis by IL-6 has, to our knowledge, not yet been sufficiently demonstrated. Given that both the classic signaling and the trans-signaling triggered by IL-6 ultimately result in the activation of the STAT3 transcription factor, we decided to study the putative effect of the IL-6/JAK/STAT3 pathway on the expression of target magnesiotropic genes (Table 1) in cell lines that possess the full membrane IL-6R. For our experiments, we chose three cell lines that are characterized by their high expression of the *IL6R* gene and the presence of a membrane form of IL-6R capable of responding to IL-6 application by triggering classic signaling, namely the HepG2 hepatocellular carcinoma cell line [43], the myeloma cell line U-266 [44], and the pancreatic carcinoma ductal origin PANC-1 cell line [45].

## 2. Results

### 2.1. Response of HEK-Blue™ IL-6 Cell Line to hIL-6

The HEK-Blue™ IL-6 reporter cell line was prepared by the stable transfection of human embryonic kidney cells HEK293 with *IL6R* and *STAT3* genes. Furthermore, the HEK-Blue™ IL-6 cell line was modified by transfection with a gene encoding STAT3-inducible secreted embryonic alkaline phosphatase (SEAP). Therefore, we could monitor the activity of the signaling pathway triggered by the application of hIL-6 by using a specific colorimetric assay.

**Figure 2:**
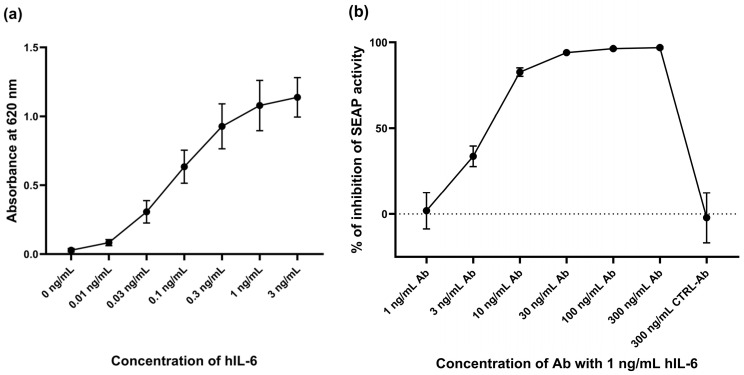
The hIL-6-specific response was quantified by a colorimetric assay by using the STAT3-inducible expression of *SEAP* and SEAP secretion into the culture medium by cells treated with various concentrations of hIL-6. (**a**) The maximum reaction to the application hIL-6 was achieved by adding hIL-6 at a concentration of 1 ng/mL (or above). (**b**) The specificity of this reaction was tested by applying a specific monoclonal anti-hIL-6-IgG antibody (Ab) at various concentrations. Anti-hIL-6-IgG antibody at a concentration of 30 ng/mL was sufficient to inhibit the signaling pathway activation, and more than 90% of the SEAP activity was suppressed. A control mouse monoclonal antibody (CTRL-Ab) was used to check antibody specificity. Values shown with standard deviation are averages of three independent experiments.

At first, we quantified the IL-6 response of the reporter cell line by using a colorimetric assay determining the activity of SEAP secreted in culture medium by human-(h)-IL-6-stimulated cells at 24 h after hIL-6 application (Figure 2a). Subsequently, we verified the specificity of the induced response by adding a monoclonal anti-hIL-6-IgG antibody (Figure 2b).

The maximum response to IL-6 stimulation occurred at hIL-6 concentrations of ≥1 ng/mL (Figure 2a). In view of this result and of the relevant literature [14,46,47,48], we decided to use hIL-6 at a concentration of 1 ng/mL, 50 ng/mL, or 100 ng/mL, in the following experiments.

### 2.2. Activation of the IL-6/JAK/STAT3 Signaling Pathway in Three Responsive Cell Lines

**Figure 3:**
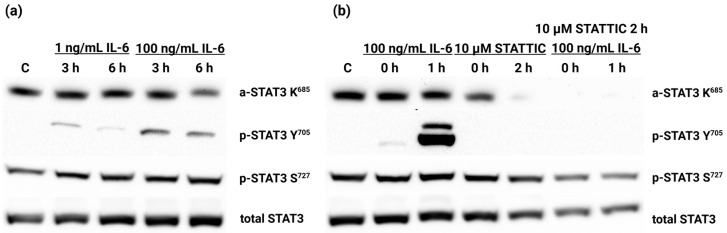
Immunodetection of post-translational modifications of the STAT3 protein triggered by the application of hIL-6 to the HEK-Blue™ IL-6 cell line. (**a**) Cells were treated with 1 or 100 ng/mL of hIL-6 for 3 or 6 h. (**b**) After 1 h of pretreatment with 10 µmol/L Stattic, 100 ng/mL hIL-6 was added to the culture medium, and the cells were incubated for another hour. The application of a Stattic inhibitor suppressed the IL-6-induced phosphorylation of Y^705^ and the subsequent acetylation of K^685^ of the STAT3 molecule.

The activation of the IL-6/JAK/STAT3 signaling pathway was monitored at the level of post-translational modifications of STAT3. The phosphorylation of STAT3 at Y^705^ by JAK leads to STAT3 dimerization and its translocation to the nucleus, where it acts as a transcription factor [18]. Furthermore, STAT3 can be phosphorylated at S^727^ by the activity of other kinases [22]. This modification stabilizes the complex of STAT3 with the transcriptional coactivator p300 [26]. The acetylation of STAT3 at K^685^ occurs in the nucleus and ensures the stability of the dimer [27]. We have monitored the phosphorylation of Y^705^ and S^727^ and the acetylation of K^685^ with specific antibodies by Western blotting (Figure 3a).

Although the phosphorylation of Y^705^ is the only post-translational modification of STAT3 that appears to be stimulated by a 3-h or 6-h treatment of the HEK-Blue™ IL-6 cell line by hIL-6, we decided to use the bioactive molecule Stattic (Tocris Bioscience, Bristol, UK), which inhibits STAT3 activation, dimerization, and nuclear localization [49]. Cells pretreated with Stattic (10 µmol/L for 1 h) and subsequently treated with hIL-6 (100 ng/mL for 1 h) showed a response to the presence of hIL-6 by the phosphorylation of Y^705^, independently of the phosphorylation of S^727^. Y^705^ phosphorylation was not detectable after the exposure of cells to the Stattic inhibitor. The acetylation of K^685^ was also affected (Figure 3b).

**Figure 4:**
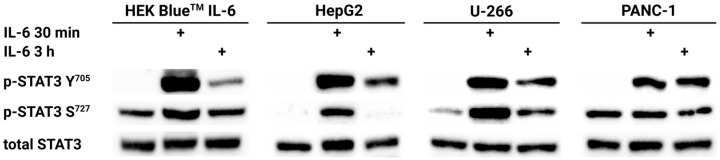
Treatment of HEK-Blue™ IL-6, HepG2, U-266, and PANC-1 cell lines with 50 ng/mL hIL-6 for 30 min or 3 h triggered STAT3 phosphorylation at Y^705^. In the case of HepG2 and U-266, S^727^ was also phosphorylated after IL-6 administration, mostly after a shorter incubation with hIL-6. Furthermore, we observed stable STAT3 phosphorylation at S^727^ in HEK Blue^TM^ IL-6 and PANC-1 cell lines, which was also present under control conditions.

Since the presence of the complete membrane form of the IL-6 receptor is essential for triggering the classic IL-6/JAK/STAT3 signaling pathway, we used cell lines characterized by the high expression of the *IL6R* gene and the presence of a membrane form of IL-6R capable of responding to soluble IL-6. To verify the ability of HepG2, U-266, and PANC-1 cell lines to respond to the induction of hIL-6 by triggering the IL-6/JAK/STAT3 pathway, cells were exposed to 50 ng/mL hIL-6 for 30 min and 3 h. Western blot analysis of the total STAT3 protein, STAT3 Y^705^, and STAT3 S^727^-phosphorylated proteins was performed. The result confirmed that the presence of hIL-6 induces STAT3 phosphorylation at Y^705^ and, in the case of the HepG2 and U-266 cell lines, even the phosphorylation of S^727^ (Figure 4).

### 2.3. Putative Changes in the Expression of Magnesiotropic Genes Triggered by hIL-6

**Figure 5:**
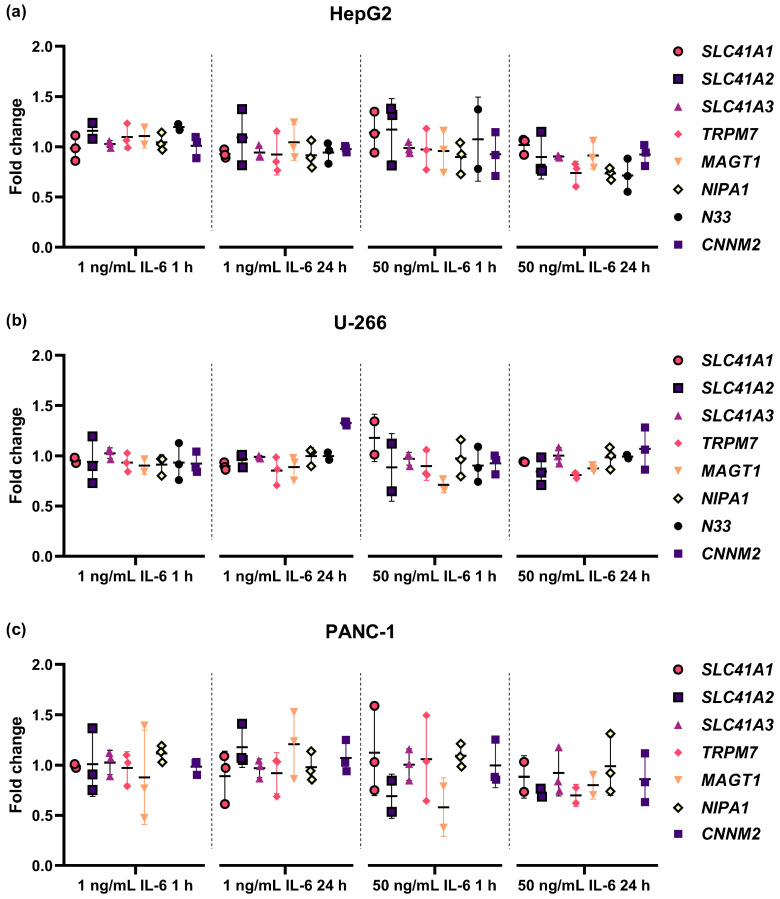
Relative expression ratios of eight analyzed magnesiotropic genes normalized to the expression rate of three reference genes (*ACTB*, *GAPDH*, *YWHAZ*). Cells were treated with hIL-6 at a concentration of 1 or 50 ng/mL for 1 or 24 h. Experiments were carried out with three cell lines, namely HepG2 (**a**), U-266 (**b**), and PANC-1 (**c**), in three independent replicates. The means, excluding outliers, are presented together with the standard deviation.

After verification of the hIL-6-sensitivity of the three cell lines, they were used for the analysis of possible changes in the expression of magnesiotropic genes after 1 or 24 h of treatment with 1 or 50 ng/mL hIL-6, directly addressing the hypothesis of the study that IL-6 treatment can influence the transcription of magnesiotropic genes. The RT-qPCR analysis of the expression of *SLC41A1*, *SLC41A2*, *SLC41A3*, *TRPM7*, *MAGT1*, *NIPA1*, *N33*, or *CNNM2* revealed any biologically relevant changes in transcription rate after normalization to the expression of three reference genes: *ACTB, GAPDH*, and *YWHAZ* (Figure 5, Supplementary Figure S1 and Table S1). A mathematical evaluation of the data obtained was conducted to determine whether any trends toward biological relevance could be observed. Although several statistically significant differences were observed in the expression of the examined magnesiotropic genes under the given conditions (Supplementary Tables S1 and S2), we do not regard these changes as biologically relevant. *TRPM6* transcription in all three cell lines studied was below the detection limit of our method. The *N33* gene had to be omitted from the analysis in PANC-1 cell line because of its weak expression. These findings suggest that IL-6 does not exert a direct regulatory effect on the transcription of magnesiotropic genes in the cell lines studied within the time frame and concentrations examined.

## 3. Discussion

Many studies have shown a correlation between increased IL-6 concentration and a lower Mg level [50,51,52]. A reduced cellular Mg^2+^ intake is also well documented as leading to an increase in the synthesis of IL-6 and to its release [12,53]. For example, a higher concentration of Mg^2+^ in the culture medium leads to the suppression of an increased expression of *IL6* by vascular smooth muscle cells under pro-inflammatory conditions; similarly, higher doses of Mg suppress the increase of pro-inflammatory IL-6 in the plasma of an animal model of chronic kidney disease [9]. Likewise, long-term dietary Mg deficiency in such animal models leads to an elevated plasma [IL-6] [42]. The TRPM7-kinase-deficient animal model, in addition to other pro-inflammatory parameters, is characterized by increased *IL6* expression accompanied by reduced intracellular [Mg^2+^] [53]. Neonatal monocytes affected by MgSO_4_ treatment decrease their production of IL-6 under constitutive and Toll-like receptor(TLR)-stimulated conditions by reducing nuclear factor kappa B (NF-κB) activation [12]. This reduction of NF-κB activity might be a consequence of the Mg^2+^-mediated inhibition of L-type calcium channels, thus preventing inflammation [54].

In contrast to the known effect of altered Mg homeostasis on IL-6 production, the influence of IL-6 on the expression of magnesiotropic genes (and the function of the corresponding proteins) remains elusive. Theoretically, IL-6 could affect Mg homeostasis at various levels, from intestinal absorption and renal excretion, through intracellular transport, to storage accumulation. Here, we have examined the influence of IL-6 via its specific JAK/STAT3 signaling pathway on the expression of various magnesiotropic genes.

Although the regulation of Mg homeostasis by IL-6 demands a thorough study, a solid body of evidence has shown the effect of IL-6 supplementation on the cellular or whole-body homeostasis of divalent cations other than Mg^2+^ [47,55,56]. For instance, a short-term application of IL-6 leads to an increase in intracellular [Ca^2+^] in rat carotid body glomus cells [55] and a reduction in reticular storage [57]. In addition to the cellular regulation of Ca homeostasis, IL-6 also affects Ca handling at the level of the entire organism. IL-6 increases the expression of the gene for the calcium-sensing receptor (CaSR), a protein that monitors and regulates Ca levels. The effect of IL-6 on gene expression regulation is even more pronounced in the case of combination with tumor necrosis factor (TNFα) [47]. However, IL-6 might also reduce gene expression, as in the case of ATPase Sarcoplasmic/Endoplasmic Reticulum Ca^2+^ Transporting 2 (*SERCA2*) [58] and Calcium Voltage-Gated Channel Subunit Alpha1 C (*Cacna1c*) [59]. Furthermore, the chronic exposure of cells to IL-6 significantly affects intracellular Ca homeostasis and changes the cellular response to external environmental stimuli [60]. The assumption can thus be made that the long-term administration of IL-6 affects the expression of genes responsible for the Ca^2+^ balance in cells and for Ca^2+^ signaling.

A typical manifestation of the inflammatory process that accompanies the acute phase response is hypoferremia and hypozincemia [61,62]. The reduced concentration of zinc in the bloodstream might result from its accumulation in the liver. This is likely due to the increased expression of the gene encoding the Zn^2+^ transporter Zip14 in hepatocytes in the presence of IL-6 [63]. In addition to *ZIP14*, an increase in the expression of genes for other Zn^2+^ transporters, namely ZnT7 and ZIP7, has also been observed after the administration of IL-6 [64]. Furthermore, *SLC39A10* expression has been shown to be regulated by JAK/STAT signaling in the presence of IL-7 [65]. IL-6 trans-signaling also triggers the accumulation of Fe^2+^ ions and ferrotoxicity in neurons, possibly leading to neurodegeneration [56].

Nevertheless, no magnesiotropic gene regulated by IL-6 is known. However, evidence exists that IL-6 can modulate intracellular [Mg^2+^]. Liu et al. have demonstrated the inhibitory effect of IL-6 on TRPM7 inward currents through both the classic and the trans-signaling cascades. The regulation of TRPM7 activity is [IL-6]-dependent and can be reversed by the inhibition of STAT3 activation [46]. However, because of the acute effect of the IL-6 application on TRPM7, we can exclude the possibility that STAT3 acts as a transcription factor regulating the expression of *TRPM7*. Notwithstanding, the empirical evidence suggests that the non-transcriptional activities of STAT3 may influence TRPM7 function [66].

A possible pleiotropic influence of IL-6 (other than at the level of transcription) on Mg homeostasis and the activity of Mg^2+^ transport mechanisms has been related to the ability of IL-6 to influence indirectly the distribution of Mg^2+^ in the cell.

The concept based on experimental evidence obtained by the research group of Abid [67] depicts the role of IL-6 signaling in mitochondrial physiology in skeletal muscle. In this model, extracellular IL-6 activates the canonical IL-6 receptor, increasing STAT3 phosphorylation at Y^705^ while reducing phosphorylation at S^727^. This destabilizes mitochondrial complex I and enhances reactive oxygen species (ROS) production. Acutely, this is demonstrated by increased mitochondrial respiration, increased mitochondrial fusion, mitochondrial biogenesis, and the further Y^705^ phosphorylation of STAT3 [67]. Chronic exposure to IL-6 might lead to a decompensated state in which ROS production is increased further but with impaired respiratory capacity and reduced signals for mitochondrial biogenesis [67]. Furthermore, the effect of the increased ROS production on the decrease of mitochondrial membrane potential, Ψ_m_ (MMP), is known [68]. Hypothetically, IL-6 might, through this complex cascade of events, inhibit the superconductive mitochondrial Mg^2+^ channel MRS2-(mitochondrial RNA splicing 2)-dependent Mg^2+^ influx into the mitochondrial matrix [69,70]. This could prevent the replenishment of mitochondrial Mg^2+^ pools and lead to further worsening of energy homeostasis, disrupting cellular Mg^2+^ balance, and altering the transcription of magnesiotropic genes [39]. Although this hypothesis has yet to be experimentally verified, a plethora of indirect evidence suggests its feasibility and potential to clarify the mechanism by which IL-6-mediated chronic inflammation influences cellular Mg homeostasis and perhaps transcription activities of genes.

The limitations of this experimental study include monitoring the expression of only a limited number of confirmed or putative magnesiotropic genes. Even within this restricted panel of nine genes, we observed differences in transcription levels between the three cell lines used (HepG2, U-266, and PANC-1). Therefore, it is clear that the transcriptional levels of these genes are cell-specific and thus, changes in the expression of these genes due to IL-6 may significantly depend on the cell type used. Furthermore, other factors, such as cytokine concentration, duration of exposure, or the use of IL-6 in combination with other inflammatory cytokines, can also have an impact.

## 4. Materials and Methods

### 4.1. Cell Cultures

In our experiments, we used four different human cell lines: IL-6 reporter line HEK-Blue™ IL-6 Cells (Invitrogen, Carlsbad, CA, USA), hepatoblastoma HepG2, multiple myeloma U-266, and pancreas carcinoma PANC-1 (all three DSMZ, Braunschweig, Germany), cultured at 37 °C in a humidified atmosphere of 5% CO_2_ and maintained in the culture media as recommended by the supplier and supplemented with 10% heat-inactivated fetal bovine serum and 1% Pen-Strep (HEK-Blue™ IL-6 Cells-DMEM 4.5 g/L glucose, HEK-Blue™ Selection (InvivoGen, San Diego, CA, USA), Normocin™ (InvivoGen, USA); HepG2-DMEM 1 g/L glucose; U-266-RPMI 1640, 1 mmol/L sodium pyruvate, 4.5 g/L glucose; PANC-1-DMEM 4.5 g/L glucose). Only cells below the 20th passage were used for experiments. Gene expression was analyzed after treatment of the cells, seeded 24 h earlier, with recombinant hIL-6 at 1, 50, or 100 ng/mL under the previously described conditions.

### 4.2. IL-6 Response Colorimetric Assay

To determine the effective concentration of hIL-6, we used the standard assay described in the protocol of the supplier of the HEK-Blue™ IL-6 Cells: 5 × 10^5^ cells per well were seeded in a 96-well plate and directly treated with hIL-6 at a concentration ranging from 0.01 to 3 ng/mL. We determined the activity of SEAP in the culture medium by using a QUANTI-Blue^TM^ solution (Invitrogen, Carlsbad, CA, USA) with the subsequent evaluation of colorimetric change being measured by using the SYNERGY H4 Microplate Reader spectrophotometer (BioTek, Winooski, VT, USA). Anti-hIL-6-IgG antibody mabg-hil6-3 (Invitrogen, Carlsbad, CA, USA) and a control mouse monoclonal antibody (Invitrogen, USA) were used to determine the specificity of the reaction.

### 4.3. Western Blot Analysis

Cells treated with 50 or 100 ng/mL for a specific time and the corresponding untreated controls were washed to remove the culture medium with Dulbecco′s Phosphate Buffered Saline without Ca^2+^ and Mg^2+^ (Sigma-Aldrich, St. Louis, MO, USA). All cells were then lysed in a lysis solution consisting of 1% CHAPS, 150 mmol/L NaCl, 15 mmol/L Tris (pH = 7.6) with HALT™ protease inhibitor cocktail (Thermo Scientific, Waltham, MA, USA) and phosphatase inhibitors (200 µmol/L Na_3_VO_4_ and 50 mmol/L NaF). After lysis at 4 °C for 20 min with constant rotation, the insoluble cell debris was removed after centrifugation at 16,000× *g* and 4 °C for 25 min. The proteins were precipitated in acetone. Protein samples were separated on 8% SDS-polyacrylamide gels under reducing conditions at a constant current of 15 mA per gel and subsequently transferred to 0.2 µm nitrocellulose membranes by semi-dry transfer at a constant voltage of 25 V within 50 min. Membranes were incubated overnight at 4 °C with the appropriate primary antibody for a particular form of STAT3. Antibodies against total STAT3 sc-8019 (1:500), phosphorylated Y^705^ sc-81523 (1:500), phosphorylated S^727^ sc-71792 (1:500) (all Santa Cruz Biotechnology, USA), or acetylated K^685^ PA5-17429 (1:1000) (Invitrogen, Carlsbad, CA, USA) were used, followed by a 1-h incubation with either anti-mouse (1:10,000) or anti-rabbit (1:20,000) horseradish peroxidase-conjugated secondary antibody (both Sigma-Aldrich, Louisville, KY, USA). After incubation with the Clarity-Western ECL substrate solution (Bio-Rad, Hercules, CA, USA), the signal was detected using the Molecular Imager Gel Doc XRS+ (Bio-Rad, Hercules, CA, USA).

### 4.4. RT-qPCR and Data Analysis

The expression of nine putative STAT3-responsive genes affecting Mg homeostasis was analyzed in HepG2, U-266, and PANC-1 cell lines. Cells in Petri dishes treated with hIL-6 were lysed directly after the removal of the culture medium, and total RNA was isolated using the RNeasy^®^ Mini Kit (Qiagen, Hilde, Germany). The quantity and purity of RNA were determined spectrophotometrically. Total RNA (1 μg) was used as a template for cDNA synthesis with the iScript™ gDNA Clear cDNA Synthesis Kit (Bio-Rad, Hercules, CA, USA) according to the manufacturer’s protocol. Subsequently, cDNA synthesized from 25 ng RNA was used as a template for a single qPCR reaction. The 10-µL reaction mixture, also containing PowerTrack^TM^ SYBR^TM^ Green Master Mix reagents (Applied Biosystems, Foster City, CA, USA) and 600 ng of each appropriate oligonucleotide for a specific gene (Table 2), was run in three technical replicates and analyzed on a ViiA7 instrument (Applied Biosystems, Foster City, CA, USA) in fast mode (Table 3), followed by melt curve analysis. We evaluated the change in the expression of genes of interest by using the efficiency-adjusted N0 method [71]. The raw fluorescence data from ViiA7 were imported into LinRegPCR v.2021.2 software [72,73]. The calculated average N0 of the gene of interest for each condition was normalized to the geometric mean N0 of three reference genes: *ACTB*, *GAPDH*, and *YWHAZ*. After normalization to control conditions without IL-6 treatment, we obtained the fold change values in three biological replicates, which were subsequently subjected to statistical analysis. Graphs were created using Prism 8 (GraphPad, San Diego, CA, USA). Data adhere to MIQE standards [74].

### 4.5. Data Analysis

Data were explored and analyzed in R version 4.4.0 (24 April 2024) [75] with the aid of the libraries (Supplementary Table S3) for data analysis. The data on fold change (FC) were summarized by mean, standard deviation (sd), the minimal value (min), lower quartile (Q1), median, and upper quartile (Q3), and the maximal value (max) for time and treatment and for each gene. Boxplot, density plot, and quantile-quantile plot were used to explore the distribution of FC across treatment, time, and gene. Outliers (*n* = 4) were identified using the Hampel filter and excluded from the subsequent analyses. A three-way ANOVA with non-constant variance was fitted to the data by the linear regression model by using the Generalized Least Squares method. From the full model (expression~gene*treatment*time), a submodel of the form (using the Wilkinson Rogers notation): expression~gene*treatment + time was selected based on the significance in ANOVA table. The selected model was subjected to the standard diagnostics by using residuals. Goodness-of-fit was quantified by the Adjusted R2. The model was used to obtain the estimated marginal means and to perform post hoc pairwise comparisons. *p* values were adjusted using the Benjamini-Hochberg correction.

## 5. Conclusions

Our work demonstrates that, in the tested cellular systems (HepG2, U-266, PANC-1), IL-6, the core cytokine of inflammation, does not regulate the expression of the tested magnesiotropic genes at the transcriptional level and, therefore, does not influence cellular Mg homeostasis through the regulation of the transcription of its major constituents (Mg^2+^ transporters and homeostatic factors). While the study did not find a significant influence of IL-6 on magnesiotropic gene transcription under the tested conditions, these findings do not exclude that the relationship may depend on additional factors such as specific cellular contexts or signaling pathways not addressed in this study. We should emphasize that the molecular and functional characteristics of the majority of the magnesiotropic genes are rather poor, and that perhaps other genes’ encoding factors playing a role in Mg homeostasis have yet to be discovered. Therefore, this work strictly reflects our current knowledge on magnesiotropic genes. Moreover, as previously mentioned, IL-6 might alter the function of Mg homeostatic factors at levels other than transcriptional level. Additionally, further detailed research on the precise role of the currently identified or putative magnesiotropic factors is necessary. It is also important to search for new factors and their regulation to better understand the maintenance of Mg homeostasis under both physiological and pathological conditions.

## Figures and Tables

**Table 1:** List of magnesiotropic factors.

Protein Name	Protein Role	References
SLC41A1	Na^+^/Mg^2+^ exchanger, regulated extrusion through plasma membrane.	[32,33]
SLC41A2	Mg^2+^ transporter, presumed localization in the plasma or organelle membrane.	[41]
SLC41A3	Na^+^/Mg^2+^ exchanger, release of Mg^2+^ from mitochondria.	[35]
TRPM6	Mg^2+^ uptake through the plasma membrane, epithelial cells of the kidney, and colon.	[30,36]
TRPM7	Mg^2+^ uptake through the plasma membrane, ubiquitously expressed.	[28]
MAGT1 & N33	Mg-homeostatic factors, integral parts of the protein N-glycosylation complex.	[37,38]
NIPA1	The Mg^2+^ transporter, with its highest abundance in brain tissue, may be important for the maintenance of the nervous system.	[39]
CNNM2	Mg-homeostatic factor, the pathogenic form of CNNM2 is associated with hypomagnesemia and epilepsy.	[40]

**Table 2:** List of oligonucleotides.

Gene	Forward Primer Sequence	Reverse Primer Sequence
*SLC41A1*	GATTCTCCTGTACATCGCAGAC	CCCCTATGAGCCAGAGAACA
*SLC41A2*	TGGTTATAAGTAGCATTGGGGGCCT	TCCTGCTAGCCTGAATGGCCA
*SLC41A3*	CACAAAGATAGTCGGTATCTGACG	GACCATGGCCAGGATGATT
*TRPM6*	GGATCTCTCTGCCCTGACTG	TTCTCTCCAGCGATCTCCAT
*TRPM7*	TGGGAAGGCTGAATATGAGG	TCGCTGTCATCCATTGTCAT
*MAGT1*	GGGATTGCTTTTGGCTGTTA	TATGGGCATATGGTGGTCCT
*NIPA1*	AACAACCCGTCCAGTCAGAG	GTAGTAGATGGCCCCGAACA
*N33*	ATGGAATGGAGTTCCAGACG	TCATTAGCTTGCCTGCACAC
*CNNM2*	TGCAGGTGATCTTCATTTCG	GCAGTGAGCACAGCAGGTAG
*ACTB*	AACGGCTCCGGCATGTGCAAG	CACATAGGAATCCTTCTGACC
*GAPDH*	CTTTGGTATCGTGGAAGGAC	TAGAGGCAGGGATGATGTTC
*YWHAZ*	CTGTAACTGAGCAAGGAGCTG	ATACTTGAGACGACCCTCCA

**Table 3:** Fast cycling conditions.

Step		Temperature	Time
	Polymerase activation	95 °C	2 min
40×	Denaturation	95 °C	5 s
	Annealing & polymerization	58 °C	30 s
	Melt curve	58–95 °C	1.99 °C/s; 15 s
		95–60 °C	1.77 °C/s; 1 min
		60–95 °C	0.075 °C/s; 15 s

## Data Availability

Data are available upon reasonable request from the first author and the corresponding author.

## Supplementary Materials

The following supporting information can be downloaded at: https://www.mdpi.com/article/10.3390/ijms252413274/s1, Figure S1. Fold change of eight analyzed magnesiotropic genes normalized to the expression rate of three reference genes (*ACTB*, *GAPDH*, *YWHAZ*). Table S1. Changes in the expression levels of the eight magnesiotropic genes in three cell lines; Table S2. Post hoc pairwise comparisons with Benjamini-Hochberg adjustment of *p* values. Table S3. Libraries used for data analysis.
