# Supplementary material for: IL-6 Does Not Influence the Expression of SLC41A1 and Other Mg-Homeostatic Factors
Source: Int J Mol Sci. 2024 Dec 11;25(24):13274. doi: 10.3390/ijms252413274 (PMC11675721; doi:10.3390/ijms252413274)
Supplement: Supplementary file 1 [file ijms-25-13274-s001.zip › ijms-3316273-supplementary.pdf]

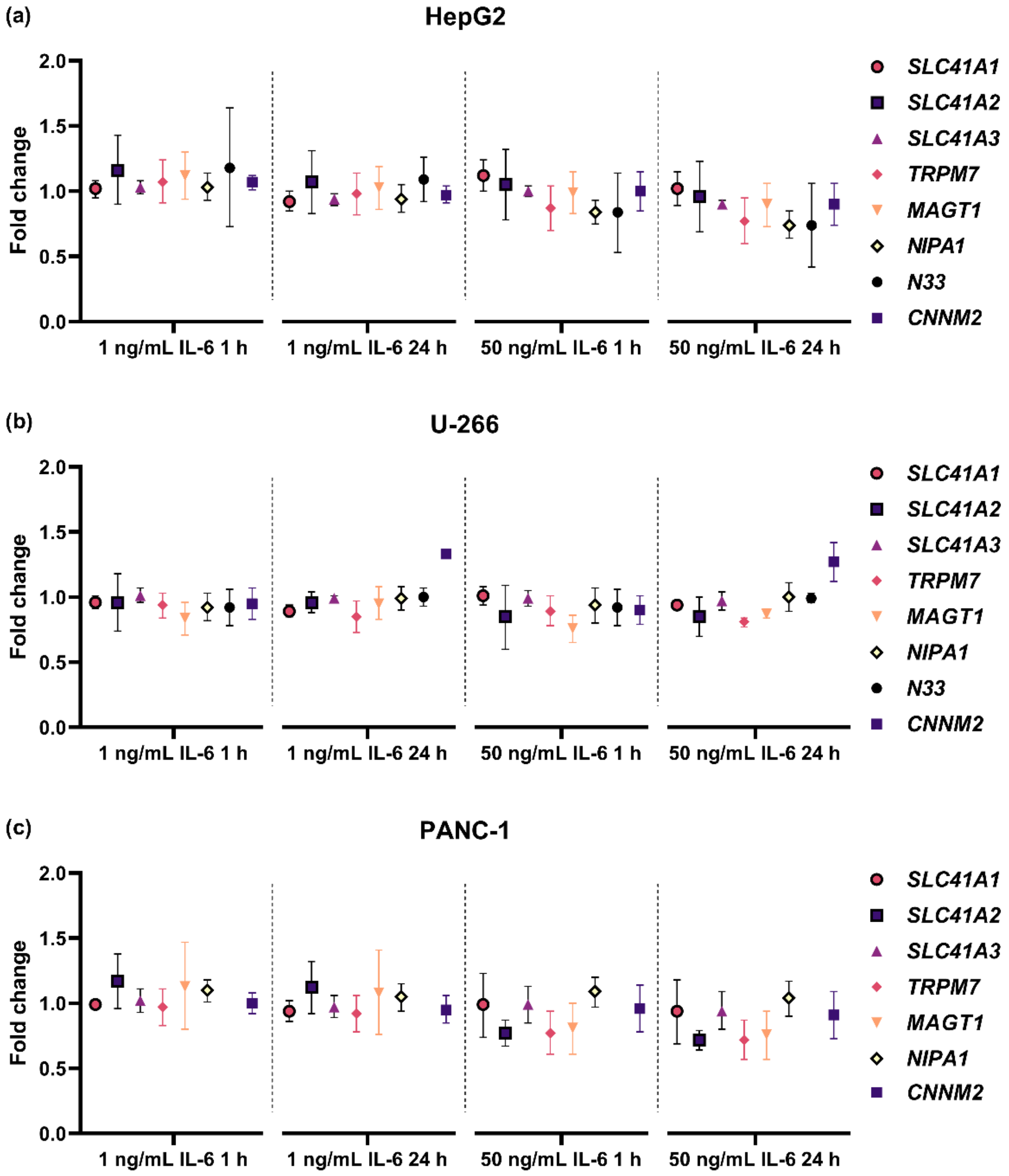

Figure S1. Fold change of eight analyzed magnesiotropic genes normalized to the expression rate of three reference genes (*ACTB*, *GAPDH*, *YWHAZ*). Cells were treated with hIL-6 at a concentration of 1 or 50 ng/mL for 1 or 24 h. Experiments were carried out with three cell lines (HepG2, U-266, and PANC-1) in three independent replicates. The estimated marginal means are displayed together with their respective upper and lower limits.

Table S1. Changes in the expression levels of the eight studied magnesiotropic genes in three cell lines.

Cell line	Gene	IL-6 concentration	Time	EMM	SE	df	Lower CL	Upper CL
HepG2	*SLC41A1*	1 ng/mL	1 h	1.02	0.03	7.66	0.95	1.08
			24 h	0.92	0.02	2.95	0.85	1.00
		50 ng/mL	1 h	1.12	0.04	4.33	1.00	1.24
			24 h	1.02	0.04	3.16	0.89	1.15
	SLC41A2	1 ng/mL	1 h	1.16	0.06	2.06	0.90	1.43

			24 h	1.07	0.07	2.38	0.83	1.31
		50 ng/mL	1 h	1.05	0.10	4.47	0.78	1.32
			24 h	0.96	0.10	4.28	0.69	1.23
	SLC41A3	1 ng/mL	1 h	1.03	0.02	3.37	0.98	1.08
			24 h	0.94	0.02	9.62	0.89	0.98
		50 ng/mL	1 h	1.00	0.02	13.97	0.96	1.04
			24 h	0.90	0.01	2.11	0.88	0.93
	TRPM7	1 ng/mL	1 h	1.07	0.06	3.99	0.91	1.24
			24 h	0.98	0.06	4.59	0.82	1.14
		50 ng/mL	1 h	0.87	0.06	4.02	0.70	1.04
			24 h	0.77	0.06	3.44	0.60	0.95
	MAGT1	1 ng/mL	1 h	1.12	0.06	3.17	0.94	1.30
			24 h	1.03	0.06	3.57	0.86	1.19
		50 ng/mL	1 h	0.99	0.06	4.80	0.83	1.15
			24 h	0.90	0.06	4.30	0.73	1.06
	NIPA1	1 ng/mL	1 h	1.03	0.04	4.23	0.93	1.14
			24 h	0.94	0.04	5.43	0.84	1.05
		50 ng/mL	1 h	0.84	0.03	4.85	0.75	0.93
			24 h	0.74	0.03	2.74	0.64	0.85
	N33	1 ng/mL	1 h	1.18	0.03	0.94	0.73	1.64
			24 h	1.09	0.03	1.77	0.92	1.26
		50 ng/mL	1 h	0.84	0.09	2.72	0.53	1.14
			24 h	0.74	0.09	2.52	0.42	1.06
	CNNM2	1 ng/mL	1 h	1.07	0.02	9.71	1.01	1.12
			24 h	0.97	0.02	2.32	0.91	1.04
		50 ng/mL	1 h	1.00	0.05	4.07	0.85	1.15
			24 h	0.90	0.05	3.34	0.74	1.06
**U-266**	***SLC41A1***	1 ng/mL	1 h	0.96	0.03	34	0.91	1.01
			24 h	0.89	0.02	34	0.85	0.94
		50 ng/mL	1 h	1.01	0.03	34	0.94	1.08
			24 h	0.94	0.00	34	0.94	0.94
	SLC41A2	1 ng/mL	1 h	0.96	0.11	34	0.74	1.18
			24 h	0.96	0.04	34	0.88	1.04
		50 ng/mL	1 h	0.85	0.12	34	0.60	1.09
			24 h	0.85	0.07	34	0.70	1.00
	SLC41A3	1 ng/mL	1 h	1.01	0.03	34	0.96	1.07
			24 h	0.99	0.01	34	0.97	1.01
		50 ng/mL	1 h	0.99	0.03	34	0.93	1.05
			24 h	0.97	0.03	34	0.90	1.04
	TRPM7	1 ng/mL	1 h	0.94	0.05	34	0.84	1.03
			24 h	0.85	0.06	34	0.73	0.97
		50 ng/mL	1 h	0.89	0.06	34	0.78	1.01
			24 h	0.81	0.02	34	0.77	0.84
	MAGT1	1 ng/mL	1 h	0.84	0.06	34	0.71	0.96
			24 h	0.95	0.06	34	0.83	1.08
		50 ng/mL	1 h	0.76	0.05	34	0.65	0.86
			24 h	0.87	0.02	34	0.84	0.91
	NIPA1	1 ng/mL	1 h	0.92	0.05	34	0.82	1.03
			24 h	0.99	0.05	34	0.90	1.08
		50 ng/mL	1 h	0.94	0.07	34	0.80	1.07
			24 h	1.00	0.06	34	0.89	1.11
	N33	1 ng/mL	1 h	0.92	0.07	34	0.78	1.06

			24 h	1.00	0.03	34	0.93	1.07
		50 ng/mL	1 h	0.92	0.07	34	0.78	1.06
			24 h	0.99	0.02	34	0.96	1.03
	CNNM2	1 ng/mL	1 h	0.95	0.06	34	0.83	1.07
			24 h	1.33	0.01	34	1.30	1.35
		50 ng/mL	1 h	0.90	0.05	34	0.79	1.01
			24 h	1.27	0.08	34	1.12	1.42
**PANC-1**	*SLC41A1*	1 ng/mL	1 h	0.99	0.01	36	0.97	1.01
			24 h	0.94	0.04	36	0.86	1.02
		50 ng/mL	1 h	0.99	0.12	36	0.74	1.23
			24 h	0.94	0.12	36	0.69	1.18
	SLC41A2	1 ng/mL	1 h	1.17	0.10	36	0.96	1.38
			24 h	1.12	0.10	36	0.92	1.32
		50 ng/mL	1 h	0.77	0.05	36	0.67	0.87
			24 h	0.72	0.04	36	0.64	0.79
	SLC41A3	1 ng/mL	1 h	1.02	0.05	36	0.93	1.11
			24 h	0.97	0.04	36	0.89	1.06
		50 ng/mL	1 h	0.99	0.07	36	0.85	1.13
			24 h	0.94	0.07	36	0.80	1.09
	TRPM7	1 ng/mL	1 h	0.97	0.07	36	0.83	1.11
			24 h	0.92	0.07	36	0.78	1.06
		50 ng/mL	1 h	0.77	0.08	36	0.61	0.94
			24 h	0.72	0.07	36	0.57	0.87
	MAGT1	1 ng/mL	1 h	1.13	0.16	36	0.80	1.47
			24 h	1.08	0.16	36	0.76	1.41
		50 ng/mL	1 h	0.81	0.10	36	0.61	1.00
			24 h	0.76	0.09	36	0.57	0.94
	NIPA1	1 ng/mL	1 h	1.10	0.04	36	1.01	1.18
			24 h	1.05	0.05	36	0.94	1.15
		50 ng/mL	1 h	1.09	0.06	36	0.97	1.20
			24 h	1.04	0.07	36	0.90	1.17
	CNNM2	1 ng/mL	1 h	1.00	0.04	36	0.92	1.08
			24 h	0.95	0.05	36	0.85	1.06
		50 ng/mL	1 h	0.96	0.09	36	0.78	1.14
			24 h	0.91	0.09	36	0.73	1.09

* The above table presents the estimated marginal means from the qPCR analysis of the expression of the eight studied magnesiotropic genes. Under most conditions, the estimated marginal means are close to a fold change of 1, indicating minimal changes in gene expression across the various experimental conditions. For a change to be considered biologically significant, we set a threshold of a fold change of 2 or 0.5, respectively. Changes beyond these thresholds are deemed to have potential biological relevance.

Table S2. Post hoc pairwise comparisons with Benjamini-Hochberg adjustment of *p* values.

Cell line	Gene	Contrast	Estimate	SE	df	t.ratio	p.value	Lower CL	Upper CL
**HepG2**	*SLC41A1*	24 h 1 ng/mL - 1 h 1 ng/mL	-0.09	0.02	15.3	-5.16	0.0003	-0.15	-0.04
		24 h 1 ng/mL - 24 h 50 ng/mL	-0.10	0.05	4.9	-2.06	0.1152	-0.30	0.11
		24 h 1 ng/mL - 1 h 50 ng/mL	-0.19	0.05	6.5	-3.78	0.0155	-0.38	0.00
		1 h 1 ng/mL - 24 h 50 ng/mL	0.00	0.05	5.9	-0.09	0.9302	-0.20	0.19
		1 h 1 ng/mL - 1 h 50 ng/mL	-0.10	0.05	4.9	-2.06	0.1152	-0.30	0.11

		24 h 50 ng/mL - 1 h 50 ng/mL	-0.09	0.02	15.3	-5.16	0.0003	-0.15	-0.04
	SLC41A2	24 h 1 ng/mL - 1 h 1 ng/mL	-0.09	0.02	15.3	-5.16	0.0003	-0.15	-0.04
		24 h 1 ng/mL - 24 h 50 ng/mL	0.11	0.12	6.5	0.95	0.4524	-0.33	0.56
		24 h 1 ng/mL - 1 h 50 ng/mL	0.02	0.12	7.2	0.16	0.8755	-0.42	0.46
		1 h 1 ng/mL - 24 h 50 ng/mL	0.21	0.12	6.3	1.73	0.2642	-0.24	0.66
		1 h 1 ng/mL - 1 h 50 ng/mL	0.11	0.12	6.5	0.95	0.4524	-0.33	0.56
		24 h 50 ng/mL - 1 h 50 ng/mL	-0.09	0.02	15.3	-5.16	0.0003	-0.15	-0.04
	SLC41A3	24 h 1 ng/mL - 1 h 1 ng/mL	-0.09	0.02	15.3	-5.16	0.0003	-0.15	-0.04
		24 h 1 ng/mL - 24 h 50 ng/mL	0.03	0.02	10.1	1.52	0.1602	-0.04	0.10
		24 h 1 ng/mL - 1 h 50 ng/mL	-0.06	0.04	12.3	-1.69	0.1602	-0.17	0.05
		1 h 1 ng/mL - 24 h 50 ng/mL	0.13	0.02	4.0	7.07	0.0044	0.04	0.21
		1 h 1 ng/mL - 1 h 50 ng/mL	0.03	0.02	10.1	1.52	0.1602	-0.04	0.10
		24 h 50 ng/mL - 1 h 50 ng/mL	-0.09	0.02	15.3	-5.16	0.0003	-0.15	-0.04
	TRPM7	24 h 1 ng/mL - 1 h 1 ng/mL	-0.09	0.02	15.3	-5.16	0.0003	-0.15	-0.04
		24 h 1 ng/mL - 24 h 50 ng/mL	0.21	0.08	7.7	2.46	0.0484	-0.09	0.50
		24 h 1 ng/mL - 1 h 50 ng/mL	0.11	0.09	9.7	1.29	0.2265	-0.18	0.40
		1 h 1 ng/mL - 24 h 50 ng/mL	0.30	0.08	7.3	3.57	0.0171	0.00	0.60
		1 h 1 ng/mL - 1 h 50 ng/mL	0.21	0.08	7.7	2.46	0.0484	-0.09	0.50
		24 h 50 ng/mL - 1 h 50 ng/mL	-0.09	0.02	15.3	-5.16	0.0003	-0.15	-0.04
	MAGT1	24 h 1 ng/mL - 1 h 1 ng/mL	-0.09	0.02	15.3	-5.16	0.0003	-0.15	-0.04
		24 h 1 ng/mL - 24 h 50 ng/mL	0.13	0.08	7.6	1.54	0.1970	-0.17	0.42
		24 h 1 ng/mL - 1 h 50 ng/mL	0.03	0.09	9.2	0.40	0.6962	-0.25	0.32
		1 h 1 ng/mL - 24 h 50 ng/mL	0.22	0.08	7.4	2.64	0.0641	-0.08	0.52
		1 h 1 ng/mL - 1 h 50 ng/mL	0.13	0.08	7.6	1.54	0.1970	-0.17	0.42
		24 h 50 ng/mL - 1 h 50 ng/mL	-0.09	0.02	15.3	-5.16	0.0003	-0.15	-0.04
	NIPA1	24 h 1 ng/mL - 1 h 1 ng/mL	-0.09	0.02	15.3	-5.16	0.0003	-0.15	-0.04
		24 h 1 ng/mL - 24 h 50 ng/mL	0.20	0.05	8.0	3.84	0.0059	0.02	0.38
		24 h 1 ng/mL - 1 h 50 ng/mL	0.10	0.06	13.0	1.81	0.0929	-0.07	0.28
		1 h 1 ng/mL - 24 h 50 ng/mL	0.29	0.05	6.9	5.68	0.0016	0.10	0.48
		1 h 1 ng/mL - 1 h 50 ng/mL	0.20	0.05	8.0	3.84	0.0059	0.02	0.38
		24 h 50 ng/mL - 1 h 50 ng/mL	-0.09	0.02	15.3	-5.16	0.0003	-0.15	-0.04
	N33	24 h 1 ng/mL - 1 h 1 ng/mL	-0.09	0.02	15.3	-5.16	0.0003	-0.15	-0.04
		24 h 1 ng/mL - 24 h 50 ng/mL	0.35	0.10	3.2	3.59	0.0391	-0.21	0.90
		24 h 1 ng/mL - 1 h 50 ng/mL	0.25	0.10	3.9	2.51	0.0672	-0.24	0.75
		1 h 1 ng/mL - 24 h 50 ng/mL	0.44	0.10	3.0	4.61	0.0371	-0.15	1.02
		1 h 1 ng/mL - 1 h 50 ng/mL	0.35	0.10	3.2	3.59	0.0391	-0.21	0.90
		24 h 50 ng/mL - 1 h 50 ng/mL	-0.09	0.02	15.3	-5.16	0.0003	-0.15	-0.04
	CNNM2	24 h 1 ng/mL - 1 h 1 ng/mL	-0.09	0.02	15.3	-5.16	0.0003	-0.15	-0.04
		24 h 1 ng/mL - 24 h 50 ng/mL	0.07	0.06	4.0	1.28	0.3219	-0.20	0.34
		24 h 1 ng/mL - 1 h 50 ng/mL	-0.02	0.06	4.9	-0.38	0.7204	-0.27	0.22
		1 h 1 ng/mL - 24 h 50 ng/mL	0.16	0.06	4.8	2.78	0.0819	-0.09	0.42
		1 h 1 ng/mL - 1 h 50 ng/mL	0.07	0.06	4.0	1.28	0.3219	-0.20	0.34
		24 h 50 ng/mL - 1 h 50 ng/mL	-0.09	0.02	15.3	-5.16	0.0003	-0.15	-0.04
**U-266**	*SLC41A1*	24 h 1 ng/mL - 1 h 1 ng/mL	-0.07	0.03	34	-2.02	0.0622	-0.16	0.03
		24 h 1 ng/mL - 24 h 50 ng/mL	-0.05	0.02	34	-2.25	0.0622	-0.11	0.01
		24 h 1 ng/mL - 1 h 50 ng/mL	-0.11	0.05	34	-2.33	0.0622	-0.25	0.02
		1 h 1 ng/mL - 24 h 50 ng/mL	0.02	0.03	34	0.74	0.4619	-0.05	0.09
		1 h 1 ng/mL - 1 h 50 ng/mL	-0.05	0.02	34	-2.25	0.0622	-0.11	0.01
		24 h 50 ng/mL - 1 h 50 ng/mL	-0.07	0.03	34	-2.02	0.0622	-0.16	0.03
	SLC41A2	24 h 1 ng/mL - 1 h 1 ng/mL	0.00	0.11	34	0.04	0.9679	-0.31	0.32
		24 h 1 ng/mL - 24 h 50 ng/mL	0.11	0.08	34	1.39	0.5227	-0.11	0.34
		24 h 1 ng/mL - 1 h 50 ng/mL	0.12	0.13	34	0.88	0.6840	-0.26	0.49

		1 h 1 ng/mL - 24 h 50 ng/mL	0.11	0.14	34	0.75	0.6840	-0.29	0.51
		1 h 1 ng/mL - 1 h 50 ng/mL	0.11	0.08	34	1.39	0.5227	-0.11	0.34
		24 h 50 ng/mL - 1 h 50 ng/mL	0.00	0.11	34	0.04	0.9679	-0.31	0.32
	SLC41A3	24 h 1 ng/mL - 1 h 1 ng/mL	-0.02	0.03	34	-0.82	0.6346	-0.10	0.05
		24 h 1 ng/mL - 24 h 50 ng/mL	0.02	0.03	34	0.64	0.6346	-0.07	0.12
		24 h 1 ng/mL - 1 h 50 ng/mL	0.00	0.03	34	-0.04	0.9710	-0.09	0.09
		1 h 1 ng/mL - 24 h 50 ng/mL	0.04	0.05	34	0.84	0.6346	-0.10	0.19
		1 h 1 ng/mL - 1 h 50 ng/mL	0.02	0.03	34	0.64	0.6346	-0.07	0.12
		24 h 50 ng/mL - 1 h 50 ng/mL	-0.02	0.03	34	-0.82	0.6346	-0.10	0.05
	TRPM7	24 h 1 ng/mL - 1 h 1 ng/mL	-0.09	0.06	34	-1.47	0.3021	-0.25	0.08
		24 h 1 ng/mL - 24 h 50 ng/mL	0.04	0.06	34	0.71	0.5803	-0.12	0.20
		24 h 1 ng/mL - 1 h 50 ng/mL	-0.04	0.10	34	-0.42	0.6745	-0.34	0.25
		1 h 1 ng/mL - 24 h 50 ng/mL	0.13	0.05	34	2.51	0.1009	-0.01	0.27
		1 h 1 ng/mL - 1 h 50 ng/mL	0.04	0.06	34	0.71	0.5803	-0.12	0.20
		24 h 50 ng/mL - 1 h 50 ng/mL	-0.09	0.06	34	-1.47	0.3021	-0.25	0.08
	MAGT1	24 h 1 ng/mL - 1 h 1 ng/mL	0.12	0.06	34	2.12	0.0963	-0.04	0.27
		24 h 1 ng/mL - 24 h 50 ng/mL	0.08	0.06	34	1.32	0.2356	-0.09	0.25
		24 h 1 ng/mL - 1 h 50 ng/mL	0.20	0.10	34	2.05	0.0963	-0.07	0.47
		1 h 1 ng/mL - 24 h 50 ng/mL	-0.04	0.07	34	-0.56	0.5822	-0.22	0.15
		1 h 1 ng/mL - 1 h 50 ng/mL	0.08	0.06	34	1.32	0.2356	-0.09	0.25
		24 h 50 ng/mL - 1 h 50 ng/mL	0.12	0.06	34	2.12	0.0963	-0.04	0.27
	NIPA1	24 h 1 ng/mL - 1 h 1 ng/mL	0.06	0.06	34	1.03	0.7437	-0.11	0.24
		24 h 1 ng/mL - 24 h 50 ng/mL	-0.01	0.06	34	-0.17	0.8636	-0.19	0.17
		24 h 1 ng/mL - 1 h 50 ng/mL	0.05	0.10	34	0.54	0.8636	-0.22	0.32
		1 h 1 ng/mL - 24 h 50 ng/mL	-0.07	0.08	34	-0.90	0.7437	-0.30	0.16
		1 h 1 ng/mL - 1 h 50 ng/mL	-0.01	0.06	34	-0.17	0.8636	-0.19	0.17
		24 h 50 ng/mL - 1 h 50 ng/mL	0.06	0.06	34	1.03	0.7437	-0.11	0.24
	N33	24 h 1 ng/mL - 1 h 1 ng/mL	0.08	0.07	34	1.12	0.5089	-0.12	0.27
		24 h 1 ng/mL - 24 h 50 ng/mL	0.01	0.04	34	0.18	0.8612	-0.10	0.11
		24 h 1 ng/mL - 1 h 50 ng/mL	0.08	0.08	34	1.00	0.5089	-0.15	0.32
		1 h 1 ng/mL - 24 h 50 ng/mL	-0.07	0.07	34	-0.97	0.5089	-0.28	0.13
		1 h 1 ng/mL - 1 h 50 ng/mL	0.01	0.04	34	0.18	0.8612	-0.10	0.11
		24 h 50 ng/mL - 1 h 50 ng/mL	0.08	0.07	34	1.12	0.5089	-0.12	0.27
	CNNM2	24 h 1 ng/mL - 1 h 1 ng/mL	0.37	0.06	34	6.19	0.0000	0.20	0.54
		24 h 1 ng/mL - 24 h 50 ng/mL	0.06	0.08	34	0.74	0.4626	-0.15	0.27
		24 h 1 ng/mL - 1 h 50 ng/mL	0.43	0.06	34	7.56	0.0000	0.27	0.59
		1 h 1 ng/mL - 24 h 50 ng/mL	-0.32	0.12	34	-2.55	0.0232	-0.66	0.03
		1 h 1 ng/mL - 1 h 50 ng/mL	0.06	0.08	34	0.74	0.4626	-0.15	0.27
		24 h 50 ng/mL - 1 h 50 ng/mL	0.37	0.06	34	6.19	0.0000	0.20	0.54
**PANC-1**	***SLC41A1***	24 h 1 ng/mL - 1 h 1 ng/mL	-0.05	0.04	36	-1.33	0.5729	-0.15	0.05
		24 h 1 ng/mL - 24 h 50 ng/mL	0.00	0.12	36	0.03	0.9754	-0.34	0.35
		24 h 1 ng/mL - 1 h 50 ng/mL	-0.05	0.14	36	-0.34	0.9754	-0.43	0.33
		1 h 1 ng/mL - 24 h 50 ng/mL	0.05	0.12	36	0.45	0.9754	-0.28	0.39
		1 h 1 ng/mL - 1 h 50 ng/mL	0.00	0.12	36	0.03	0.9754	-0.34	0.35
		24 h 50 ng/mL - 1 h 50 ng/mL	-0.05	0.04	36	-1.33	0.5729	-0.15	0.05
	SLC41A2	24 h 1 ng/mL - 1 h 1 ng/mL	-0.05	0.04	36	-1.33	0.1910	-0.15	0.05
		24 h 1 ng/mL - 24 h 50 ng/mL	0.40	0.11	36	3.75	0.0012	0.10	0.70
		24 h 1 ng/mL - 1 h 50 ng/mL	0.35	0.12	36	3.03	0.0068	0.03	0.67
		1 h 1 ng/mL - 24 h 50 ng/mL	0.45	0.11	36	4.07	0.0012	0.14	0.76
		1 h 1 ng/mL - 1 h 50 ng/mL	0.40	0.11	36	3.75	0.0012	0.10	0.70
		24 h 50 ng/mL - 1 h 50 ng/mL	-0.05	0.04	36	-1.33	0.1910	-0.15	0.05
	SLC41A3	24 h 1 ng/mL - 1 h 1 ng/mL	-0.05	0.04	36	-1.33	0.5729	-0.15	0.05
		24 h 1 ng/mL - 24 h 50 ng/mL	0.03	0.08	36	0.35	0.7890	-0.19	0.25

	24 h 1 ng/mL - 1 h 50 ng/mL	-0.02	0.08	36	-0.27	0.7890	-0.25	0.21
	1 h 1 ng/mL - 24 h 50 ng/mL	0.08	0.09	36	0.84	0.7890	-0.18	0.33
	1 h 1 ng/mL - 1 h 50 ng/mL	0.03	0.08	36	0.35	0.7890	-0.19	0.25
	24 h 50 ng/mL - 1 h 50 ng/mL	-0.05	0.04	36	-1.33	0.5729	-0.15	0.05
TRPM7	24 h 1 ng/mL - 1 h 1 ng/mL	-0.05	0.04	36	-1.33	0.2052	-0.15	0.05
	24 h 1 ng/mL - 24 h 50 ng/mL	0.20	0.10	36	1.95	0.1173	-0.08	0.48
	24 h 1 ng/mL - 1 h 50 ng/mL	0.15	0.11	36	1.29	0.2052	-0.17	0.47
	1 h 1 ng/mL - 24 h 50 ng/mL	0.25	0.10	36	2.45	0.1153	-0.03	0.53
	1 h 1 ng/mL - 1 h 50 ng/mL	0.20	0.10	36	1.95	0.1173	-0.08	0.48
	24 h 50 ng/mL - 1 h 50 ng/mL	-0.05	0.04	36	-1.33	0.2052	-0.15	0.05
MAGT1	24 h 1 ng/mL - 1 h 1 ng/mL	-0.05	0.04	36	-1.33	0.1910	-0.15	0.05
	24 h 1 ng/mL - 24 h 50 ng/mL	0.33	0.19	36	1.76	0.1738	-0.19	0.85
	24 h 1 ng/mL - 1 h 50 ng/mL	0.28	0.19	36	1.45	0.1910	-0.26	0.81
	1 h 1 ng/mL - 24 h 50 ng/mL	0.38	0.19	36	2.00	0.1738	-0.15	0.90
	1 h 1 ng/mL - 1 h 50 ng/mL	0.33	0.19	36	1.76	0.1738	-0.19	0.85
	24 h 50 ng/mL - 1 h 50 ng/mL	-0.05	0.04	36	-1.33	0.1910	-0.15	0.05
NIPA1	24 h 1 ng/mL - 1 h 1 ng/mL	-0.05	0.04	36	-1.33	0.5729	-0.15	0.05
	24 h 1 ng/mL - 24 h 50 ng/mL	0.01	0.07	36	0.14	0.8923	-0.19	0.21
	24 h 1 ng/mL - 1 h 50 ng/mL	-0.04	0.08	36	-0.51	0.8923	-0.26	0.18
	1 h 1 ng/mL - 24 h 50 ng/mL	0.06	0.08	36	0.73	0.8923	-0.17	0.29
	1 h 1 ng/mL - 1 h 50 ng/mL	0.01	0.07	36	0.14	0.8923	-0.19	0.21
	24 h 50 ng/mL - 1 h 50 ng/mL	-0.05	0.04	36	-1.33	0.5729	-0.15	0.05
CNNM2	24 h 1 ng/mL - 1 h 1 ng/mL	-0.05	0.04	36	-1.33	0.5729	-0.15	0.05
	24 h 1 ng/mL - 24 h 50 ng/mL	0.05	0.10	36	0.47	0.7722	-0.22	0.32
	24 h 1 ng/mL - 1 h 50 ng/mL	0.00	0.11	36	-0.04	0.9644	-0.31	0.30
	1 h 1 ng/mL - 24 h 50 ng/mL	0.10	0.10	36	0.96	0.6884	-0.18	0.37
	1 h 1 ng/mL - 1 h 50 ng/mL	0.05	0.10	36	0.47	0.7722	-0.22	0.32
	24 h 50 ng/mL - 1 h 50 ng/mL	-0.05	0.04	36	-1.33	0.5729	-0.15	0.05

* The above table presents the results of post hoc tests conducted after the performance of a three-way ANOVA on the qPCR data. Post hoc pairwise comparisons were used to identify statistically significant differences between individual groups. The *p* values were adjusted using the Benjamini-Hochberg correction. Despite the presence of several statistically significant differences in the expression of the examined magnesiotropic genes under the given conditions, we do not consider these changes to be biologically relevant.

Table S3. Libraries used for data analysis.

Almeida A, Loy A, Hofmann H (2018). _ggplot2 Compatible Quantile-Quantile Plots in R_, volume 10 number 2. <https://doi.org/10.32614/RJ-2018-051>.
Clarke E, Sherrill-Mix S, Dawson C (2023). _ggbeeswarm: Categorical Scatter (Violin Point) Plots_. R package version 0.7.2, <https://CRAN.R-project.org/package=ggbeeswarm>.
Fox J, Weisberg S (2019). _An R Companion to Applied Regression_, Third edition. Sage, Thousand Oaks CA. <https://socialsciences.mcmaster.ca/jfox/Books/Companion/>.
Fox J, Weisberg S, Price B (2022). _carData: Companion to Applied Regression Data Sets_. R package version 3.0-5, <https://CRAN.R-project.org/package=carData>.
Goode K, Rey K (2019). _ggResidpanel: Panels and Interactive Versions of Diagnostic Plots using 'ggplot2'_. R package version 0.3.0, <https://CRAN.R-project.org/package=ggResidpanel>.
Lenth R (2024). _emmeans: Estimated Marginal Means, aka Least-Squares Means_. R package version 1.10.2, <https://CRAN.R-project.org/package=emmeans>.
Lüdecke D (2024). _sjPlot: Data Visualization for Statistics in Social Science_. R package version 2.8.16, <https://CRAN.R-project.org/package=sjPlot>.
Ogle DH, Doll JC, Wheeler AP, Dinno A (2023). _FSA: Simple Fisheries Stock Assessment Methods_. R package version 0.9.5, <https://CRAN.R-project.org/package=FSA>.

Pinheiro J, Bates D, R Core Team (2023). _nlme: Linear and Nonlinear Mixed Effects Models_. R package version 3.1-164, <https://CRAN.R-project.org/package=nlme>. Pinheiro JC, Bates DM (2000). _Mixed-Effects Models in S and S-PLUS_. Springer, New York. doi:10.1007/b98882 <https://doi.org/10.1007/b98882>.

R Core Team (2024). _R: A Language and Environment for Statistical Computing_. R Foundation for Statistical Computing, Vienna, Austria. <https://www.R-project.org/>.

Wickham H (2016). _ggplot2: Elegant Graphics for Data Analysis_. Springer-Verlag New York. ISBN 978-3-319-24277-4, <https://ggplot2.tidyverse.org>.

Wickham H, François R, Henry L, Müller K, Vaughan D (2023). _dplyr: A Grammar of Data Manipulation_. R package version 1.1.4, <https://CRAN.R-project.org/package=dplyr>.

Zhu H (2024). _kableExtra: Construct Complex Table with 'kable' and Pipe Syntax_. R package version 1.4.0, <https://CRAN.R-project.org/package=kableExtra>.
